# How does reproduction account for dairy farm sustainability?

**DOI:** 10.1590/1984-3143-AR2023-0066

**Published:** 2023-08-04

**Authors:** Jaciara Diavão, Abias Santos Silva, Anna Luiza Lacerda Sguizzato, Camila Sousa da Silva, Thierry Ribeiro Tomich, Luiz Gustavo Ribeiro Pereira

**Affiliations:** 1 Embrapa Gado de Leite, Juiz de Fora, MG, Brasil

**Keywords:** fertility, genetic selection, methane emission, methane intensity, milk yield

## Abstract

Sustainability - the new hype of the 21^st^ century has brought discomfort for the government and society. Sustainable agriculture is essential to face our most concerning challenges: climate change, food security, and the environmental footprint, all of which add to consumers' opinions and choices. Improvements in reproductive indexes can enhance animal production and efficiency, guaranteeing profit and sustainability. Estrus detection, artificial insemination (AI), embryo transfer (ET), estrus synchronization (ES), and multiple ovulations are some strategies used to improve animal reproduction. This review highlights how reproductive strategies and genetic selection can contribute to sustainable ruminant production. Improved reproductive indices can reduce the number of nonproductive cows in the herd, reducing methane emissions and land use for production while preserving natural resources.

## Introduction

Sustainability - the new hype of the 21^st^ century has brought discomfort for the government and society. But is this topic a novelty in research and politics areas?

The concept of sustainability was first addressed in forestry near the 17^th^ and 18^th^ centuries -with the idea the never harvest more than the forest could yield in new cycles (Wiersum, 1995). However, it was only in 1987, when the United Nations World Commission on Environment and Development (WCED) published the Brundtland Report, that the term 'sustainable development' became popular and was defined as "development that meets the needs of the present without compromising the ability of future generations to meet their own needs". After that, agendas and declarations were built to guide 'sustainable development', but all of society did not accept the idea. Later, in the mid-1990s, the concept was brought into evidence again, gathering researchers' and politicians' attention (Purvis et al., 2019).

Sustainable agriculture is essential to face our most concerning challenges: climate change, food security, and the environmental footprint, all of which are added to consumers' opinions and choices. According to the United States Department of Agriculture (USDA, 2023), greenhouse gas (GHG) emissions from agriculture accounted for 11.2% of total United States of America (USA) emissions in 2020, where 5.6% is due to direct nitrous oxide, 4.2% to direct methane, 0.8% to direct carbon dioxide, and 0.6% to electricity-related. However, in 2016, Brazilian agriculture contributed 33.2% to total GHG emissions in Brazil (Brasil, 2023), evidencing the distinction on GHG emissions between countries in respect to the proportion of agriculture-based economics.

In addition, food derived from animal products (i.e.: dairy and beef) provides essential nutrients for the human diet. Thus, over the years, animal production has increased and adapted to feed the world population; however, ruminant production has contributed to GHG emissions, mainly due to enteric methane (CH_4_).

**Figure 1:**
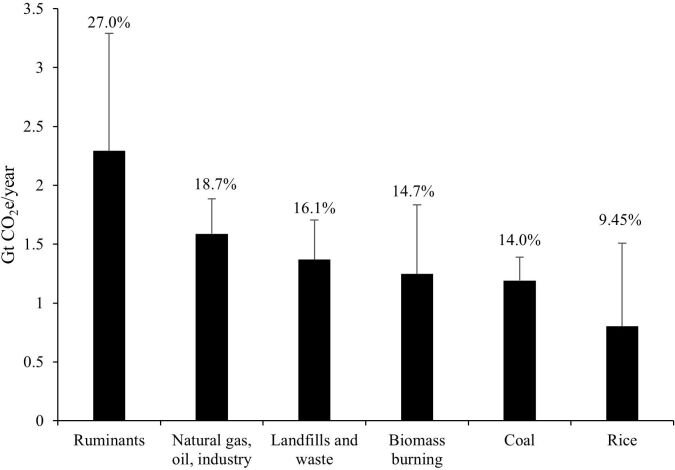
Estimated anthropogenic methane emission from major sources (Adapted from Ripple et al., 2014). The percentage over the bars denotes the percentage of each item on global GHG emission.

Methane is an abundant non-CO_2_ GHG with a shorter atmospheric lifespan, around nine years, and its reduction allows more rapid benefits for climate change (Ripple et al., 2014). The total GHG emissions from global livestock are 7.1 Gigatonnes (Gt) of carbon dioxide equivalent (CO_2_-eq) per year, representing 14.5% of all anthropogenic GHG emissions. From the 7.1 Gt CO_2_-eq, 44% of emissions are methane (CH_4_), 29% as nitrous oxide (N_2_O), and 27% as CO_2_ (FAO, 2023). There are distinct anthropogenic sources of CH_4_ (ruminants, fossil fuel industry, landfills, biomass burning, and rice production); however, ruminants are the largest source (Figure 1; Ripple et al., 2014). Moreover, CH_4_ emission intensities vary from one commodity to another. The highest levels of CO_2_-eq in livestock are produced by beef (around 300 kg CO_2_-eq/kg of protein produced), followed by small ruminants (beef and milk; 165 and 112 kg CO_2_-eq/kg of protein produced, respectively) and cow milk, chicken and pork product, which are at the bottom of emission intensity list (below 100 CO_2_-eq/kg of protein produced) (FAO, 2023).

To better address the importance of ruminants for climate change, first, we need to understand how they participate in GHG emissions. Ruminant digestion is a process of enteric fermentation in a multichambered stomach (Ripple et al., 2014), where ruminant microbes can convert plant carbohydrates to energy to benefit them and the animal (Knapp et al., 2014). In the reticulorumen and hindgut, carbohydrates are hydrolyzed by microbial enzyme activity - sugars are fermented to volatile fatty acids producing reducing equivalents (i.e., metabolic hydrogen). This metabolic hydrogen is then converted to H_2_ by hydrogenase-expressing bacterial species and H_2_ is converted to CH_4_ by methanogenic archaea. This is an essential mechanism since H_2_ can negatively impact carbohydrate degradation, microbial growth, and microbial protein synthesis (Knapp et al., 2014). Thus, it is imperative to focus on mechanisms to mitigate CH_4_ production by ruminants, such as feeding management and nutrition, rumen modifiers, and an increase in animal production through genetics and reproductive approaches (Knapp et al., 2014).

Improvements in reproductive indices can enhance animal production and efficiency, guaranteeing profit and sustainability (Hufana-Duran and Duran, 2020). Estrus detection, artificial insemination (AI), embryo transfer (ET), estrus synchronization (ES), and multiple ovulations are some strategies used to improve animal reproduction. Efficient reproduction is vital for dairy cows due to their high milk yields since low reproductive indices can increase days open, implying a more extended period in an unproductive phase (Pinedo et al., 2020). Furthermore, genetic selection associated with improved reproductive characteristics can promote sustainable livestock and decrease CH_4_ emissions by 10 to 15% (Garnsworthy, 2004). Therefore, this review highlights how reproductive strategies and genetic selection can contribute to sustainable ruminant production.

## Effect of calving intervals on greenhouse gases emissions

Dairy production comprises gestation cycles, calving, lactation, and a dry period preceding the next calving (Lehmann et al., 2016). Traditional dairy systems have managed cows to calve once a year (e.g., 12-month calving interval). This reproductive strategy is based on the idea that early conception benefits the production economy, which arose from 1960s studies showing that annual milk production was maximized by calving intervals between 12 and 13 months (Speicher and Meadows, 1967; Louca and Legates, 1968).

To achieve the 12-month of calving interval, the first insemination will occur when production levels are still high and a positive energy balance is yet to be re-established, increasing the risk of metabolic disorders and failed conception (Browne et al., 2015). Such conditions have made current dairy systems question the annual calving interval as an ideal practice. Moreover, because calving intervals are closely related to the number of calves and replacement heifers in the herd and the efficiency of milk production (Lehmann et al., 2019), recent research has focused on the role of calving intervals on GHG emissions. Mitigation strategies for GHG emissions from livestock have been pointed out as a critical part of climate obligations (Wall et al., 2012).

Wall et al. (2012) examined the effects of three lactation length scenarios (305, 370, and 440 days) on GHG emissions using United Kingdom dairy herd data. The tested lactation lengths were equivalent to the conventional annual calving target, the UK's average calving interval (12.3 months), and an 18-month calving interval. The authors estimated that longer calving intervals required fewer milking cows and replacements to maintain milk yield levels; nonetheless, CO_2_ equivalent (CE)/farm per year increased by 157 t when calving intervals were extended from 12 to 18 months. In this study, the annual herd milk yield remained constant, and the numbers of cows and replacements were allowed to vary to maintain yields for each lactation-length scenario.

When the number of cows in the herd was kept constant and calving intervals were manipulated through different timings of first insemination, Lehmann et al. (2019) reported decreases in carbon footprint (by up to 8.2% per annual cow) by extending calving intervals from 13 to 18 months due to less feed production and enteric fermentation. Similarly,Browne et al. (2015) reported lower total emissions and emissions intensity (t CO_2_e/t milk fat plus protein) for 18-month calving intervals compared to annual calving.

Several authors have advocated the extension of calving intervals and lactation in dairy cows (Lehmann et al., 2014, 2016; Sehested et al., 2019; Burgers et al., 2021). The possibility of reducing GHG emissions through longer calving intervals is mainly attributed to more lactation days and fewer dry days per cow per year (if the dry period length remains unchanged), and fewer calves and replacement heifers (reducing replacement rate per year; Lehmann et al., 2016). The GHG related to feed use by youngstock are accounted for in the milking herd; therefore, by reducing the number of youngstock, longer calving intervals could possibly aid in mitigating GHG emissions by reducing herd feed use per kilogram of milk produced and GHG emissions from animals not contributing to production (Lehmann et al., 2019; Sakatani, 2022).

Although the efficacy of extending calving intervals for mitigation of GHG emissions is still under debate, Kok et al. (2019) observed a 1.0% and 1.7% increase in GHG (CO_2eq_/t of milk fat plus protein) from heifers and cows when lactation was extended in two months and four months, respectively, but emissions were similar to baseline calving interval (mean of 390 days for primiparous and multiparous cows) or even reduced when lactation persistency or the lifespan of cows was increased. These results suggest that lactation persistency and production level (e.g., primiparous, or multiparous cows) may play a role in GHG emitted from cows managed under longer calving intervals.

## Estrus detection and GHG as a tool for sustainability

More attention to cows' reproduction and technological strategies adopting can result in efficient performance, guaranteeing profitability and sustainability (Hufana-Duran and Duran, 2020). In addition, estrus detection is an essential factor affecting reproductive performance, and failure to detect it or misdiagnosis can result in significant economic losses (Senger, 1994).

The traditional and most used estrus detection method is the farm staff's direct observation (Palmer et al., 2010), resulting in efficiency below 50% up to 90% (Roelofs et al., 2010). However, estrus detection is a usual problem of dairy farms, mainly due to the labor required (Mayo et al., 2019) for cows’ observation and the occurrence of short periods of estrus in high-producing dairy cows (Wiltbank et al., 2006), resulting in economic losses by $360 per missed estrus (De Vries, 2006).

Several devices for the automation of estrus detection have been developed to face the low rate of estrus detection (Firk et al., 2002). The use of pedometers, chin-ball markers, heat-mount detectors, devices that measure vaginal or milk temperature, and devices that measure the electrical impedance of the genitalia or vaginal mucus and radiotelemetry (Brehme et al., 2008; Duran et al., 2015) are examples. Results from studies indicate a considerable potential to detect estrus with more precision to improve detection rates and reduce error rates. In addition, estrus detection can reduce the environmental impact by reducing the number of nonproductive animals in the farms (Sakatani, 2022).

The efficiency of estrus detection and the time to the beginning of breeding after calving influenced the cost of production and methane emissions (Archer et al., 2015). For an average UK herd (126 cows and 7.353 annual milk yield per cow), this saved at least £50 per cow and a 3.6% reduction in methane emissions per liter of milk when the estrus synchronization of first insemination was used and compared with breeding based on observed estrus. So, estrus synchronization can contribute to reducing GHG emission.

## Artificial insemination and GHG

Artificial insemination (AI) is essential to improve herds' genetic efficiency (Hufana-Duran and Duran, 2020). The genetic advance achieved with artificial insemination can increase milk production without expanding the number of animals in dairy herds (Gifford and Gifford, 2013); thus, indirectly, AI can enhance the system's sustainability. The adoption of AI, mainly in Brazil, is related to using other production systems as farm-housed cows (Santos et al., 2021), reducing production areas while preserving the natural resources.

According to Hristov et al. (2013a), assisted reproductive technologies, such as AI, have a high relative effectiveness in mitigating non-CO_2_ GHG emissions (Table 1). The improvement in fertility can reduce the number of unproductive animals kept on farms and the number of replacement heifers needed. Moreover, reducing culling rates from 35 to 30% may reduce whole-herd enteric CH_4_ emissions by 3.1% when the age at first calving is around 26 months (Knapp et al., 2014).

**Table 1:** Reproductive management strategies offering non-CO_2_ greenhouse gas mitigation opportunities (Adapted from Hristov et al., 2013a).

**Category**	**Species**	**Relative effectiveness**	**Input required to achieve desired effect**
Genomic selection for fertility	All ruminants and swine	Medium	High
Artificial insemination	All ruminants and swine	High	Moderate or high
Hormonal synchronization	All ruminants and swine	Medium	High
Embryo transfer	All ruminants and swine	High	High

**Figure 2:**
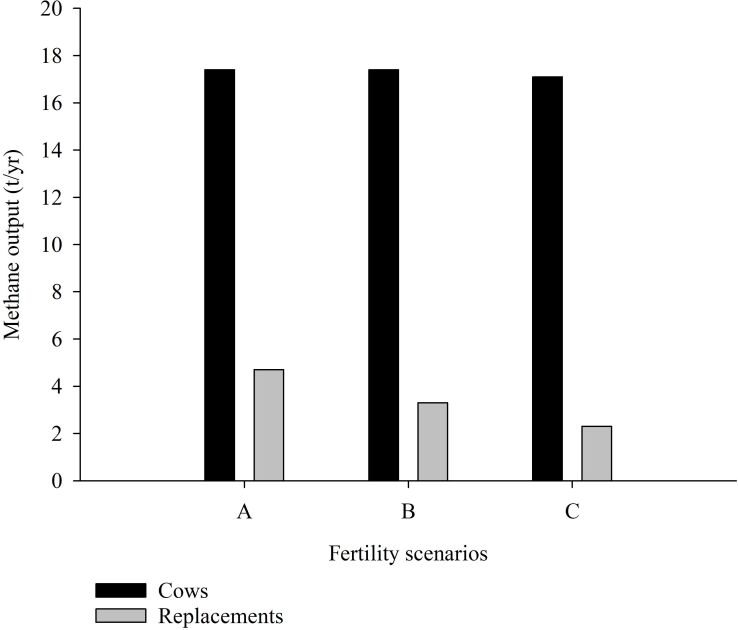
Annual methane output per 100 cows in dairy herds with no milk quota and a mean annual milk yield of 6000 kg per cow, and with current levels of fertility (A) with 78 days to first insemination, 50% of estrus detection rate, 38% of conception rate to first AI and 37% conception rate to subsequent AI; 1995 levels (B) with 72 days to first insemination, 55% of estrus detection rate, 47% of conception rate to first AI and 46% conception rate to subsequent AI or ideal levels (C) with 70 days to first insemination, 70% of estrus detection rate, 65% of conception rate to first AI and 60% conception rate to subsequent AI. Adapted from Garnsworthy (2004).

Garnsworthy (2004) also observed that fertility scenarios guided by AI would result in different CH_4_ outputs for cows and replacement heifers (ton/yr; Figure 2). Therefore, enhancement of fertility levels was likely to reduce CH_4_ emissions by 24% and ammonia emissions by 17% (Table 2).

**Table 2:** Fertility scenarios to increase reproduction and the impact on CH_4_ or ammonia emissions when comparing current fertility levels to desired levels result in reductions of 24% and 17% for CH_4_ and ammonia emissions, respectively

**Item**	**Fertility scenario**		
	**A** *	**B** **†**	**C** **‡**
First insemination (days)	78	72	70
Estrus detection rate (%)	50	55	70
Conception rate to first AI (%)	38	47	65
Conception rate to subsequent AI (%)	37	46	60

* A: current levels of fertility; ^†^B: levels of fertility in 1995; ^‡^C: desired levels of fertility. Adapted from Garnsworthy (2004).

## Embryo transfer and farm sustainability

The ET started to be developed in farm dairy cows in the 1940s and 1950s (Rowson, 1951), consisting of the transfer of a viable embryo produced *in vivo* from a donor cow or produced *in vitro* after follicular aspiration to the uterine horn of a receiving cow. From this technique, it is possible to produce several embryos of superior cows, and the introduction of *in vitro* fertilization allowed to multiply the number of embryos produced, enhancing the positive effects of embryo transfer on genetic gain, and resulting in greater milk production (Lohuis, 1995).

As discussed earlier, enhancing the number of high-producing dairy cows enables the reduction or elimination of low-producing and non-producing cows in the dairy farm; it can mean a reduction of CH_4_ intensity, mainly by the increase of conception rate and herd’s genetic gain when ET is used (Hristov et al., 2013b). Furthermore, ET is a prime strategy to improve the fertility of heat-stressed high-producing dairy cows, increasing the pregnancy rate by 80.8% compared to the prostaglandin plus estrus technique (Baruselli et al., 2020).

## Genetic improvement and GHG

For many years, livestock was blamed for the rise in GHG emissions. Over time, strategies such as genetic selection (Sypniewski et al., 2021) were implemented to reduce CH_4_ production (Króliczewska et al., 2023).

The heritability for CH_4_ traits is moderate, ranging from 0.12 to 0.45 (Breider et al., 2019; López-Paredes et al., 2020; Króliczewska et al., 2023). Furthermore, a high heritability (rg = 0.94) between daily CH_4_ production and CH_4_ intensity (de Haas et al., 2011) suggests that selecting for CH_4_ will result in lower CH_4_ units per milk produced (Kamalanathan et al., 2023) as described in temperate conditions studies (Table 3).

**Table 3:** CH_4_ reduction by genetic selection during ten years of study in temperate conditions reported by several authors

**Study**	**Breed**	**Number of cows**	**Feeding-system**	**Unit**	**CH_4_ reduction (%)**
de Haas et al. (2011)	Holstein	488	TMR†	CH_4_ production‡	11 to 26
Moate et al. (2015)	ND*	ND	Pasture-based	CH_4_ intensity§	13.3
Kandel et al. (2018)	Holstein	58412	ND	CH_4_ intensity	15
González-Recio et al. (2020)	Holstein	64	ND	CH_4_ intensity	8
López-Paredes et al. (2020)	Holstein	1501	ND	CH_4_ intensity	15
de Haas et al. (2021)	Holstein	15000	TMR	CH_4_ intensity	13
Lahart et al. (2021)	Holstein	230	Pasture-based	CH_4_ intensity	10
Richardson et al. (2022)	ND	ND	ND	CH_4_ intensity	7.84

* ND: not determined; ^†^ TMR: total mixed ration; ^‡^CH_4_ production: g/d; ^§^CH_4_ intensity: g CH_4_/kg of milk yield.

Genetic selection is a powerful strategy for reducing CH_4_ emissions. CH_4_ intensity can be reduced by 1.25% per year by genetic selection (de Haas et al., 2021). These metrics have been incorporated as a goal in breeding programs, allowing for a reduction of 0.021 mg/L in five generations (Calderón-Chagoya et al., 2021).

Because CH_4_ production is a natural final compound of metabolism in ruminants, as milk yield or dry matter intake (DMI) rises, so does CH_4_ production also increase (Lahart et al., 2021; Fresco et al., 2023) due to more availability of free-N_2_ in the rumen (Króliczewska et al., 2023). Moreover, CH_4_ production is positively correlated to DMI (R^2^ = 0.44; *P* < 0.001), and milk yield (R^2^ = 0.37; *P* < 0.001) (Min et al., 2022).

Although CH_4_ production increases as milk yield increases due to genetic selection (Hossein‑Zadeh, 2022), the main should be on CH_4_ intensity (g of CH_4_ per unit of milk yield). Reducing CH_4_ at the expense of milk yield, DMI, or sacrificing economic gains should be avoided (Richardson et al., 2022; Króliczewska et al., 2023).

High-producing dairy cows can reduce GHG intensity. Lahart et al. (2021) compared the top 5% cows to a group representative of the national average genetic merit and showed that elite cows reduced GHG intensity and enhanced N efficiency. Interestingly, this study also evaluated three feeding systems (low grass allowance; high grass allowance; and high concentrate) and found that a high concentrate diet had greater GHG due to growing, manufacturing, and transportation of the additional concentrate used, indicating that other factors, other than animal model, must be considered.

**Figure 3:**
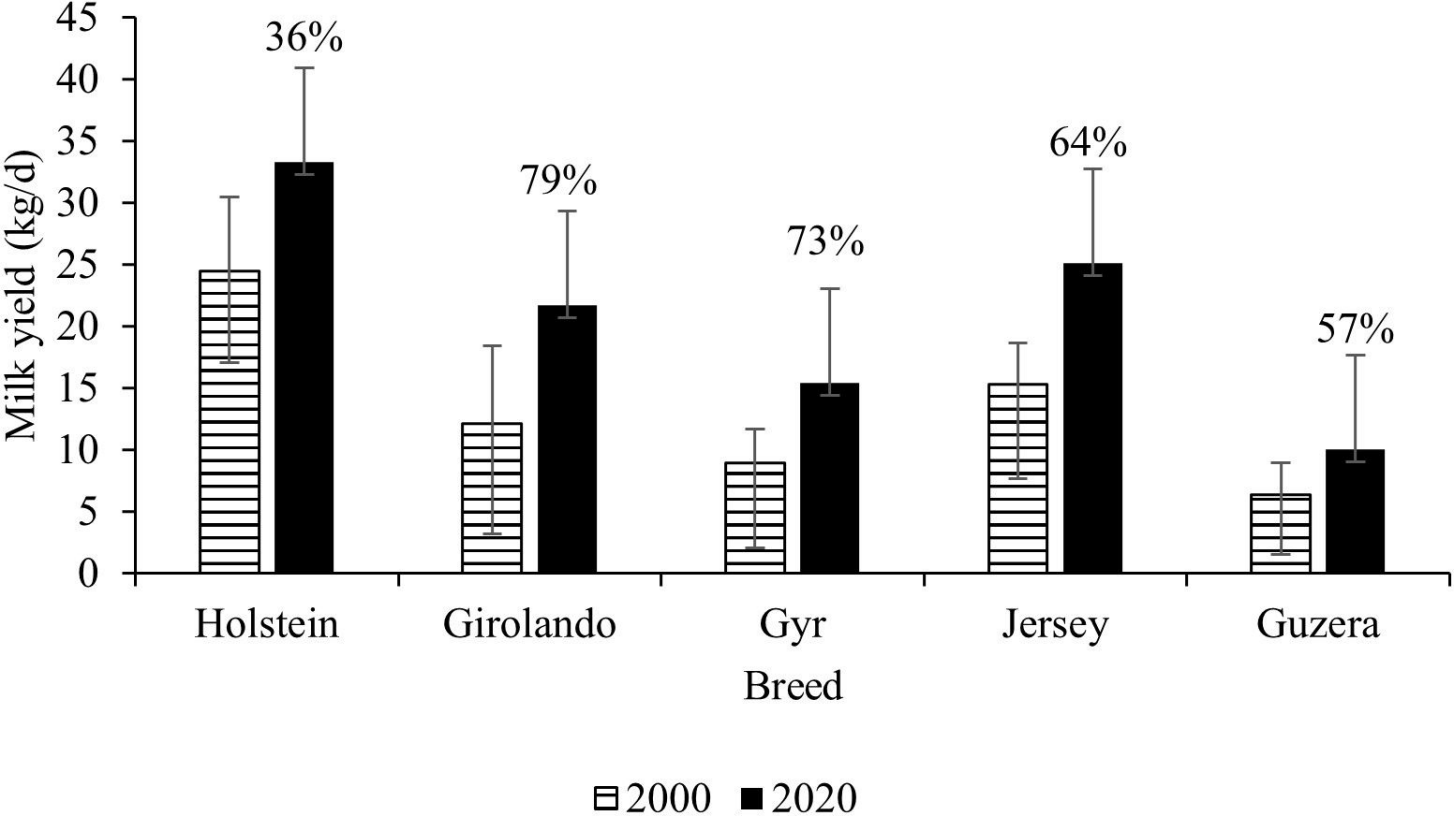
The effect of genetic selection on milk yield in Brazilian breeding programs during a twenty-year period. The percentage numbers over the bars represent the percentage of increase on milk yield from years 2000 to 2020 for each dairy breed.

**Figure 4:**
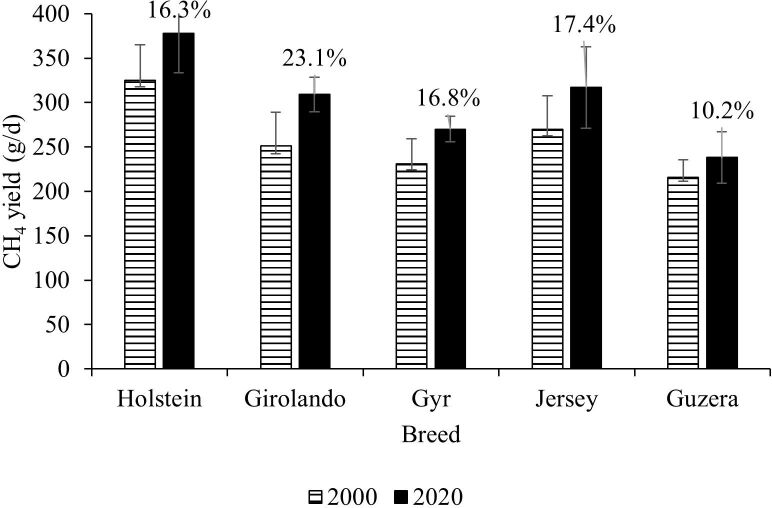
The effect of genetic selection on CH_4_ yield in Brazilian breeding programs during a twenty-year period. The percentage numbers over the bars represent the percentage of increase on CH_4_ yield from years 2000 to 2020 for each dairy breed.

**Figure 5:**
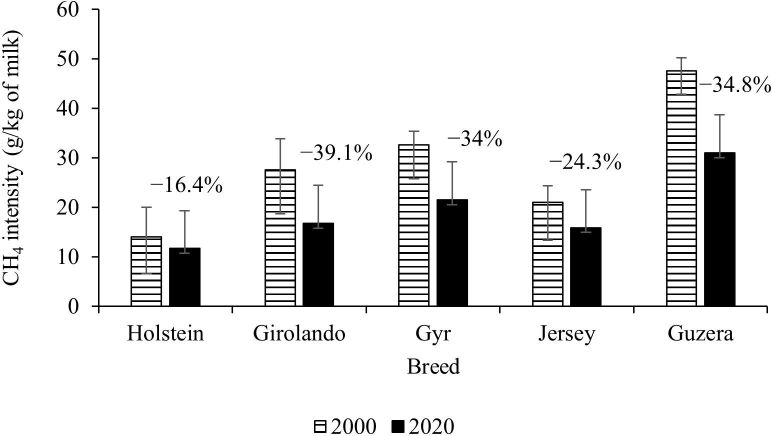
The effect of genetic selection on CH_4_ intensity in Brazilian breeding programs during a twenty-year period. The percentage numbers over the bars represent the percentage of decrease on CH_4_ intensity from years 2000 to 2020 for each dairy breed.

Breeding programs have traditionally been focused on boosting milk yield (Negri et al., 2021). Brazil, like other countries, plans to reduce GHG emissions by 30% by 2030, with the primary goal of reducing emission intensities (Willett et al., 2019). Data from primarily crossbred cows in Brazil revealed that genetic breeding programs resulted in increases in milk yield (Figure 3), CH_4_ yield (Figure 4), and reduction of CH_4_ intensity (Figure 5) during the last 20 years (Cairo, 2023 forthcoming).

This descriptive data set included 590,000 lactations from Holstein cows; 270,598 lactations from Girolando (Holstein x Gyr); 100,861 lactations from Gyr cows; 44,184 lactations from Jersey cows and 10,116 lactations from Guzera cows (Cairo, 2023 forthcoming). All breeds increased milk yield, especially Girolando (+79%) and Gyr cows (+73%). Fertility improvements (Bragança and Zangirolamo, 2018), culling rate (De Vries and Marcondes, 2020; Różańska-Zawieja et al., 2021), feeding management (Różańska-Zawieja et al., 2021), mortality reduction (Yanga and Jaja, 2021), and age to first calving (Eastham et al., 2018) can explain these findings.

As reported by Cairo (2023 forthcoming), the improvement in milk yield each year was 0.383 kg, despite Girolando and Jersey's cows increasing milk yield by over 0.5 kg per year. Similarly to other worldwide breeding program (Zhang et al., 2019), CH_4_ production increased by 16.7% in Brazil. However, the CH_4_ intensity was reduced by 0.82, 1.95, 1.70, 1.21, and 1.74% per year for Holstein, Girolando (Holstein x Gyr), Gyr, Jersey, and Guzera, respectively (Cairo, 2023 forthcoming).

CH_4_ intensity has recently emerged as a viable measure for genetic selection (Kandel et al., 2018). As a result, it is better to have fewer cows producing more milk, diluting the CH_4_ in the final product, rather than having more cows producing less CH_4_, but also less milk (de Haas et al., 2021). Furthermore, milk yield is positively correlated with CH_4_ production, indicating that caution is required when the goal of genetic selection is lower CH_4_ production (Breider et al., 2019). So, genetic selection appears to be a strategy to reducing GHG emissions and improving sustainability (Hossein‑Zadeh, 2022; González-Recio et al., 2020).

## Conclusions

Improved reproductive indices can reduce the number of nonproductive cows in the herd, reducing CH_4_ emissions and land use for production while preserving natural resources. Only genetic selection as an approach for dairy farm sustainability may reduce CH_4_ emissions by more than 1% per year.

## Data Availability

The data that support the findings of this study are available from the corresponding author upon reasonable request.
